# Uncovering the Relationship Between Bullying, Social Communication Challenges, and the Emergence of Mental Health Problems Among Saudi Children with Autism Spectrum Disorder

**DOI:** 10.3390/children12101387

**Published:** 2025-10-14

**Authors:** Mahmoud Abdelwahab Khedr, Nada Alqarawi, Rasha Mohammed Hussein

**Affiliations:** 1 College of Nursing, University of Hafr Albatin, Hafr Albatin 39921, Saudi Arabia; 2 King Salman Center for Disability Research, Riyadh 11614, Saudi Arabia; 3 Department of Psychiatric and Mental Health, and Community Health, College of Nursing, Qassim University, Buraydah 51452, Saudi Arabia; n.alqarawi@qu.edu.sa (N.A.); rm.ahmed@qu.edu.sa (R.M.H.)

**Keywords:** autism spectrum disorder, bullying, social communication challenges, mental health, Saudi Arabia

## Abstract

**Highlights:**

**What are the main findings?**

- Positive correlations were found between bullying and both social communication challenges (r = 0.306, *p* < 0.001) and emotional problems (r = 0.247, *p* = 0.002).
- Social communication challenges significantly mediate the relationship between bullying and mental health issues, indicating that difficulties in communication exacerbate the effects of bullying.

**What is the implication of the main finding?**

- There is an urgent need for interventions focused on enhancing social communication skills among children with ASD to help mitigate the impact of bullying.
- Regular mental health assessments should be implemented for children with ASD to identify and address emotional and behavioral difficulties early.
- Anti-bullying initiatives should be culturally tailored to specific social dynamics and attitudes in Saudi Arabia.

**Abstract:**

**Background/Objective:** Children with ASD are particularly vulnerable to bullying, which may exacerbate mental health issues. This study aims to explore the intricate relationships between bullying, social communication challenges, and the emergence of mental health problems among Saudi children with ASD. Additionally, it examines the mediating role of social communication challenges in these associations. **Methods:** A cross-sectional study was conducted with 150 children diagnosed with ASD at the Abdullah Altamimi Centre. Data were collected using the Child–Adolescent Bullying Scale, Social Communication Questionnaire, and Strengths and Difficulties Questionnaire. The data collection period lasted for three months, from the beginning of December 2024 to the end of February 2025. **Results:** The mean child–adolescent bullying score was 46.37, indicating prevalent bullying experiences. The mean Strengths and Difficulties Questionnaire score was 21.35, revealing significant emotional and behavioral difficulties. Positive correlations were found between bullying and social challenges (r = 0.306, *p* < 0.001) and emotional problems (r = 0.247, *p* = 0.002). Mediation analysis indicated that social communication challenges significantly mediated the relationship between bullying and strengths and difficulties scores (*p* < 0.001). **Conclusions:** Bullying significantly affects the mental health of Saudi children with ASD, highlighting the need for targeted interventions to enhance social communication skills and mitigate the impacts of bullying. These findings underscore the need to address these challenges within the cultural context of Saudi Arabia to enhance the well-being of this vulnerable population.

## 1. Introduction

Autism spectrum disorder (ASD) is generally identified as a neurodevelopmental condition manifested by distinctive social communication patterns and a persistent, diverse nature [1], associated with constrained, monotonous, or stereotypical conduct [2]. The diagnosis can be recognized as early as 18 to 48 months of life; unique symptoms can be observed in contrast to typical development, along with other delays or developmental issues during this period [3]. The global prevalence of ASD in children is approximately 1 in 160, while in Saudi Arabia, it stands at about 1 in 167 [4]. Children with autism possess unique assets and challenges, such as anxiety, hyperfocus, sensory perception differences, and special interests [5].

Children and adolescents with ASD are susceptible to bullying, which is defined as intentional, aggressive behavior that is repeated over time and involves a power imbalance between the aggressor and the victim [6]. Bullying is typically noticeable by aggressive conduct that is intentional and repetitive [7]. It manifests in multiple forms, including physical, verbal, social, property damage, or sexual aggression [8]. It persists even among children with high-functioning ASD or Asperger’s syndrome [9,10].

Correspondingly, Adams et al. (2016) declared that autistic students in mainstream contexts are more likely to be ignored, purposely excluded by their peers, victimized for having intense areas of interest, and/or prompted to exhibit social or emotional responses [11]. The earlier longitudinal study suggested that bullying victimization may be a factor in the development of mental health challenges, which refers to a range of emotional and behavioral issues that impact a child’s well-being, such as anxiety, depression, and conduct disorders [12]. This connection between bullying and mental health is particularly concerning, as the escalation in anxiety or depression rates linked to bullying victimization was markedly more pronounced among autistic children compared to their non-autistic counterparts [13].

Challenges in social communication are a hallmark of ASD. The social communication characteristics of individuals with ASD, including verbal and nonverbal communication, are contingent upon their age and cognitive and linguistic development. A range of symptoms may be identified, including an absolute absence of speech, mild language delays, and deficits in language reception, as well as echolalia [14].

Children on the autistic spectrum often struggle with social communication skills, which encompass a wide range of abilities [15]. Social communication serves as a comprehensive term, as articulated by Kossyvaki (2018, p. 7) [16], encompassing (1) social interaction, which is characterized by a reciprocal exchange of information along with relational elements such as friendships and relationships [17], sense of belonging and acceptance, loneliness and feelings of isolation, and (2) communication, which is explained as an interactive process whereby partners share information through many modalities, including words, gestures, facial expressions, and body language [18].

The neurodiversity paradigm posits that neurological differences, such as those seen in ASD, should be recognized as part of human diversity rather than as deficits to be cured [14]. This perspective challenges traditional views and emphasizes the need for inclusive environments that accommodate diverse communication styles and social interactions [18]. By adopting this paradigm, the current study aims to highlight the strengths and challenges faced by individuals with autism, thereby informing interventions that celebrate neurodiversity rather than pathologizing it.

Individuals diagnosed with ASD encounter obstacles in verbal and nonverbal communication, social skills, and language domains [19]. These obstacles result in learning difficulties and language delays [18]. In Saudi Arabia, cultural perceptions of disability play a crucial role in shaping the experiences of children with ASD. These attitudes can result in stigma and social isolation, which can intensify the difficulties these children encounter, especially regarding bullying and mental health issues [4].

Park et al. (2020) reported that the challenges associated with ASD, such as obstacles with social interactions, communication, and behavior, can result in substantial challenges in school, learning, employment, autonomy in life, social relationships, familial strain, and vulnerability to victimization and bullying [9]. A substantial correlation has been identified between the level of severity of ASD and the likelihood of being bullied [20]. Furthermore, the fact that children with ASD are ill-prepared for social interaction, particularly in the establishment and maintenance of peer relationships, and are at a higher risk of being victimized [21] underscores the necessity for the context in which they are raised to establish mechanisms that will allow them to engage equally in society [22].

Given the pervasive nature of bullying among children with ASD, it is essential to understand the broader implications for their mental health. In a global context, numerous studies have highlighted the pervasive nature of bullying among children with ASD, establishing a clear link between bullying victimization and adverse mental health outcomes. Research has shown that children with ASD face a higher risk of social isolation and mental health issues compared to their neurotypical peers, making them more vulnerable to bullying [9,21].

Mental health issues in children adversely affect all aspects of childhood development and are especially associated with emotional and behavioral challenges. Among children, the most common mental health problems include both outward manifestations, such as hyperkinetic disorder and conduct disorder, and internal manifestations, such as depression and anxiety disorders [23].

A scoping review assessing depression in autistic individuals aged 3–19 years stated that the causal relationship between social difficulties and depressive symptoms remains ambiguous and may be bidirectional. Increased awareness of social differences may heighten vulnerability to depression, whereas depressive symptoms may diminish social motivation to communicate with others [24]. This complexity is further illustrated by the phenomenon of camouflaging, where individuals on the autism spectrum often articulate that concealing their autistic characteristics serves as a strategy to mitigate prejudice and bullying [25].

While the link between bullying, ASD, and mental health is well-established globally, the sociocultural context of Saudi Arabia presents unique factors influencing these relationships. Cultural attitudes toward disability can shape the experiences of children with ASD, particularly regarding social acceptance and stigma. For instance, children may face different societal expectations and pressures that can affect their mental health and experiences with bullying [26].

In the context of Saudi Arabia, while the link between bullying, ASD, and mental health is recognized, there is a significant scarcity of research specifically addressing these issues. Recent studies indicate that the prevalence of ASD has risen significantly in Saudi Arabia, with estimates showing a notable increase over the past decade, reflecting both better awareness and improved diagnostic practices [26,27]. Cultural attitudes toward disability can shape the experiences of children with ASD, particularly regarding social acceptance and stigma. Recent studies indicate that societal expectations may influence the prevalence of bullying and its psychological impacts on children with ASD in Saudi Arabia [27].

### Significance of the Study

Previous studies have demonstrated associations between bullying victimization and ASD symptoms, including social and communication difficulties [9,25]. However, there is a significant gap in research focusing specifically on the Saudi context. This lack of localized studies limits our understanding of how cultural perceptions and social dynamics may alter the patterns of bullying and its consequences for mental health in children with ASD [27]. Addressing this gap is crucial for developing effective interventions tailored to the cultural context of Saudi Arabia. The current study seeks to explore the intricate relationships between these factors, offering insights into the mental health challenges encountered by the ASD population in Saudi Arabia.

In reference to Saudi Arabia’s Vision 2030, which aims to enhance the quality of life for all segments of the population [28], it is imperative to develop comprehensive, efficacious interventions that cater to the specific needs of individuals with ASD and their families. By examining the mediating role of social communication challenges in these associations, we hypothesize that bullying and social communication difficulties are linked to the emergence of mental health problems among Saudi children with autism spectrum disorders.

## 2. Methods

### 2.1. Study Design

The descriptive research design employed in the current study was a cross-sectional approach that adhered to the STROBE guidelines, given the time-specific nature of the data analysis.

### 2.2. Study Setting

The study was conducted at the Abdullah Altamimi Centre in Unaizah City, Qassim District; it is a branch of a nonprofit organization specializing in rehabilitation. This institution renders a wide range of services to support individuals and families. These include educational and therapeutic interventions, such as physical, occupational, psychological, and behavioral therapies; skill development; speech therapy; professional rehabilitation; family counseling; initiatives aimed at social and functional empowerment through training for labor market integration and family empowerment. The researchers selected this location and institution because of their convenience and the accessibility of the data.

### 2.3. Study Population

All children aged five years or older who met the inclusion criteria and whose parents or custodians had provided written consent to participate and were willing to take part in the study were eligible to enroll in the study. The children must have a disorder duration of at least two years and be able to comprehend and respond. The mother or instructor was requested to complete the questionnaire if the youngster was under 12 years old and unable to answer due to communication limitations. We excluded children with additional diagnoses, including neurodegenerative disorders or conditions that impede participation, along with those whose parents did not provide written consent or complete the questionnaire.

### Sample Size Calculation

The EPI INFO (Version 7.2.7) program was used to estimate the sample size [29], considering the parameters of a population consisting of 240 children diagnosed with ASD, an anticipated prevalence rate of 50%, a margin of error of 5%, and a confidence level of 95%. Therefore, the minimum sample size necessary was established to be 148 children with ASD.

**Figure 1:**
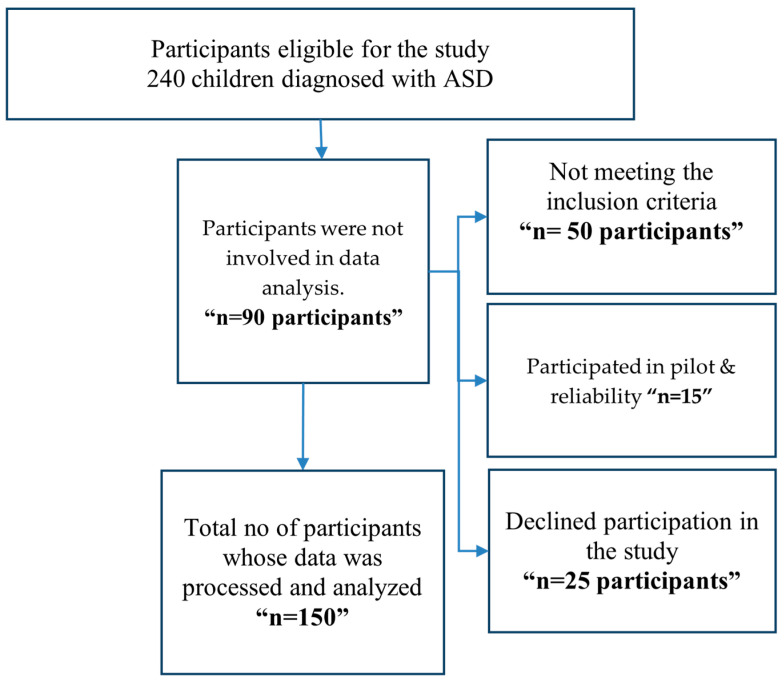
Flow chart of participants’ recruitment process.

Initially, 240 individuals with ASD were interviewed. Fifty participants were excluded because they did not meet the inclusion criteria. A total of 190 participants remained eligible for analysis. Furthermore, 15 subjects from the pilot stage of the study had been eliminated from the final sample of participants. Moreover, 25 individuals refused to take part. The study eventually comprised 150 participants, surpassing the previously established minimum sample size. The recruitment process was structured and systematic to ensure that the final sample was pertinent and appropriate for the research objectives, as verified in Figure 1.

### 2.4. Data Collection Methods

#### 2.4.1. Part I: Sociodemographic Characteristics

Researchers planned to collect sociodemographic data from study participants after an extensive review of pertinent literature. The survey comprised questions aimed at assembling sociodemographic and clinical information, focusing on variables such as age, gender, family history, age of onset for the child, duration of the disorder, presence of comorbid conditions, and kinds of treatment.

#### 2.4.2. Part II: The Child and Adolescent Bullying Scale (CABS)

Strout et al. (2018) developed the CABS, a 22-item instrument designed to identify teens who are susceptible to bullying [30]. Among the twenty elements related to bullying exposure, one pertains to the frequency of bullying, and the other relates to the presence of a perceived power differential between the adolescent and the perpetrator. The CABS score calculation excludes the two frequency and power differential-related criteria. Another study provides detailed information about the procedures used to create the CABS items [31]. This item response category spans from strongly agreeing (5) to strongly disagreeing (1). The range of total scores is 20 to 100, where higher cumulative scores indicate greater exposure to bullying throughout childhood or adolescence. Convergent and construct validity were verified by translating the CABS into Arabic through a standard forward–backwards translation process, ensuring both conceptual and linguistic equivalence. The translated version was appraised by experts for content validity and pilot-tested with a participant sample to assess clarity and reliability, achieving a standardized Cronbach’s alpha of 0.97. Furthermore, the Cronbach’s alpha of 0.929 in the previous study indicated that the instrument is a reliable and valid measure [32]. The diagnostic performance of CABS demonstrated distinguished strength relative to existing tools, with a sensitivity of 84%, specificity of 65%, and an area under the receiver operating characteristic curve (AUROC) of 0.74 (95% CI: 0.69–0.80) [28].

#### 2.4.3. Part III: Social Communication Questionnaire SCQ

The SCQ is a 40-item instrument to detect Autism Spectrum Disorder (ASD) symptoms. The SCQ in Arabic was applied and verified by Aldosari et al. (2019) to assess the behavior and interactions of children in social situations [33]. The 40 questions are binary, with answers of “Yes” or “No”. Items 2–40 are used for scoring purposes, whereas item 1 is used solely to determine if the participant can communicate in brief phrases or sentences. Consequently, the overall score falls between 0 and 39. The presence of anomalous behavior is assigned a score of 1, while its absence is assigned a score of 0. The cutoffs of 15 and 22 have been suggested to select individuals who are likely to have a broader or restricted form of ASD, as indicated by the original validation study conducted on a clinical sample [34]. Later epidemiological studies have indicated a cutoff of 12 to enhance SCQ achievements in population-based samples [35]. The internal consistency of the previous analysis was exceptional (alpha = 0.92) [33].

#### 2.4.4. Part IV: Strengths and Difficulties Questionnaire (SDQ)

SDQ was employed to measure mental health symptoms in ASD children. The 25-item teacher and parent form of the Strengths and Difficulties Questionnaire (SDQ) was used to obtain each informant’s appraisal of four problem domains/subscales and one pro-social domain/subscale, with each domain comprising five items [36,37,38]. The problem subscales include emotional symptoms, behavioral issues, inattention/hyperactivity, and peer problems. The SDQ scores each item on a 3-point ordinal scale, with zero representing ‘not true’, one representing ‘somewhat true’, and two representing ‘unquestionably true’. Higher scores indicate more significant issues, with the exception of pro-social behavior, where a greater score denotes more positive behavior. The total difficulty score of the SDQ is calculated by aggregating the scores from the four subscales of problem behavior. The SDQ score for total difficulties ranges from 0 to 40, while the subscale scores range from 0 to 10. In clinical and neurodivergent populations, it has been found to be a useful dimensional indicator of psychopathology [39,40,41,42]. The trustworthiness of the SDQ domains was found to be good in the earlier study [43].

### 2.5. Pilot Study

Upon receiving official approval from the appropriate authorities. Ten percent of the eligible individuals participated in a pilot study to assess the validity, reliability, objectivity, and transparency of the research instruments. The Arabic versions of the instruments were assessed by five mental health and psychiatric nursing experts for their cultural appropriateness, content validity, thoroughness, and item clarity in relation to Saudi culture. The subjects evaluated in the pilot study were not incorporated into the overall sample size. Completing the questionnaire usually takes 20 to 30 min. The internal robustness of the study instruments was assessed using Cronbach’s alpha. Their reliability was evident, as evidenced by the scores of 0.862 for tool CABS, 0.795 for SCQ, and 0.879 for tool SDQ.

### 2.6. Data Collection

Data collection commenced after obtaining ethical approval. On the planned follow-up day, the researchers visited both male and female sections of the Abdullah Altamimi Centre. To establish rapport, clarify the study’s objectives, and confirm participants’ consent to engage voluntarily, the researchers approached each eligible child individually based on the pre-established inclusion criteria. The researchers subsequently conducted interviews with each child, their mother, and/or teacher to gather data regarding the measured constructs. The questionnaire was anticipated to require approximately 20 to 30 min to complete. The data collection period lasted three months, from 1 December 2024 to 28 February 2025.

### 2.7. Ethical Considerations

The study received authorization from the Regional Research Ethics Committee and was authorized to proceed (607/46/6644). The administration of the Abdullah Altamimi Centre provided approval upon being informed of the study’s objectives and methodologies. To ensure confidentiality, participants were assured that their identities would not be disclosed to any individual or organization. Before completing the questionnaire, each participant signed a written authorization form. The participants were neither financially compensated nor coerced to participate in this study. The survey participants exhibited an awareness of their freedom of choice and their right to decline participation or disregard it entirely. Data privacy, voluntary participation, and anonymity were all respected in the study in accordance with the Declaration of Helsinki [44].

### 2.8. Statistical Analysis

IBM Corp. was used to gather, tabulate, and statistically analyze all of the data. This was published in 2015. IBM SPSS Statistics, Version 23.0 for Windows. Armonk, New York: IBM Corporation. Numbers and percentages were used to convey qualitative data, whereas means ± SD and medians (ranges) were used to describe quantitative data. The Chi-square test was used to compare the proportions of categorical variables. The association between several research variables was evaluated using Pearson’s correlation coefficient to assess the relationship between the study variables. Every test was two-sided. A statistically significant *p*-value was less than 0.05, while a statistically insignificant *p*-value was more than 0.05.

The association between the bullying score, the strength and difficulties score, and the social communication problems score was determined using mediation analysis, which supports a model framework wherein the independent variable, serving as a predictor variable (CABS score), is connected to the dependent variable (SDQ) via an intermediary variable functioning as a mediator (SCQ). The path coefficients in the mediation model are the beta (β)coefficients of the regression models that link the variables that were considered. To determine the significance of the mediating effect, the c bootstrap approach (with 1000 iterations) was used.

## 3. Results

Table 1 provides a detailed overview of the sociodemographic characteristics of 150 Saudi children diagnosed with Autism Spectrum Disorder (ASD). The age distribution indicates that the majority of the children are between 5 and 10 years old, accounting for 55.3% of the sample, with a mean age of 9.20 years. Gender representation shows a significant predominance of males (73.3%), which aligns with existing research on the higher prevalence of ASD in boys. The data on the age at which the children were diagnosed reveals that most (75.3%) were identified between the ages of 2 and 4 years, emphasizing the importance of early detection and intervention. The duration of the disorder indicates that many children (76.7%) have been living with ASD for 5 to 10 years, suggesting a need for ongoing support.

Regarding treatment, all participants received some form of intervention, with the majority undergoing psychiatric or behavioral therapy (68.7%) and speech treatment (28.7%). This is encouraging, as it highlights the accessibility of therapeutic services for these children. The presence of comorbid conditions is relatively low, with only 13.3% of the children reporting additional disabilities, primarily categorized under “other.” Notably, the data also reveal concerning trends in bullying; 56% of children reported being “not much” bullied, while 16.7% experienced more severe bullying situations. The perceived power dynamics in bullying scenarios show that the majority (81.3%) felt a moderate power differential between themselves and their bullies, which could significantly impact their emotional well-being.

Table 2 presents a descriptive analysis of various study variables related to Saudi children with ASD, offering insights into bullying, social communication challenges, and mental health symptoms. The Child Adolescent Bullying Scale (CABS) indicates a mean total score of 46.37, suggesting that bullying experiences are prevalent among these children, with a mean percent score of 32.96 reflecting the extent of this impact. Social communication challenges are evident, with a mean score of 17.45 and a mean percent score of 44.73, indicating significant difficulties in social interactions. The Strengths and Difficulties Questionnaire (SDQ) reveals a mean total score of 21.35, highlighting various emotional and behavioral difficulties, particularly in emotional problems (mean score of 10.14 and 50.70 percent), which is notably high. Pro-social behaviors are relatively low, suggesting challenges in social engagement.

Table 3 presents the correlations among various studied variables related to bullying, social communication challenges, and mental health symptoms in 150 Saudi children with ASD. The analysis reveals significant positive correlations between bullying and social communication challenges (r = 0.306, *p* < 0.001), indicating that higher levels of bullying are associated with increased difficulties in social communication. Furthermore, bullying is positively correlated with conduct problems (r = 0.188, *p* = 0.021) and emotional problems (r = 0.247, *p* = 0.002), suggesting that children experiencing bullying may also face more significant behavioral and emotional difficulties.

The correlations among the Strengths and Difficulties Questionnaire (SDQ) subscales provide additional insights into the relationships between these variables. Social communication challenges demonstrate a strong positive correlation with emotional problems (r = 0.336, *p* < 0.001) and peer problems (r = 0.308, *p* < 0.001), highlighting that difficulties in social communication are closely linked to emotional distress and challenges in peer relationships. Additionally, conduct problems and emotional problems are highly correlated (r = 0.532, *p* < 0.001), suggesting a significant overlap between behavioral issues and emotional challenges. The overall SDQ score shows strong positive correlations with all variables, particularly emotional problems (r = 0.894, *p* < 0.001), emphasizing the pervasive impact of bullying and social communication challenges on the mental health of children with ASD.

Table 4 presents the results of a multivariate linear regression analysis examining the factors that influence the Strengths and Difficulties Questionnaire (SDQ) scores in 150 Saudi children with Autism Spectrum Disorder (ASD). The analysis identifies both the Child Adolescent Bullying Scale (CABS) and social communication challenges as significant predictors of SDQ scores. Specifically, CABS has an unstandardized coefficient (B) of 0.069 and a standardized coefficient (Β) of 0.196, indicating that higher levels of bullying are associated with increased emotional and behavioral difficulties (t = 2.430, *p* = 0.016). Similarly, social communication challenges have a substantial impact, with a coefficient of 0.261 and a Beta of 0.248, indicating a strong association with SDQ scores (t = 3.069, *p* = 0.003). The model explains 13% of the variance in SDQ scores (R^2^ = 0.130) and is statistically significant (F = 10.977, *p* < 0.001), underscoring the importance of addressing bullying and communication difficulties in interventions designed to improve mental health outcomes for children with ASD.

**Figure 2:**
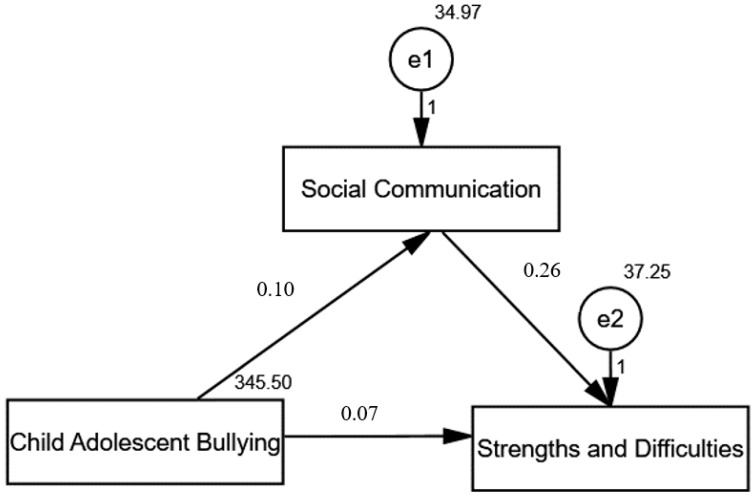
Model fit parameters CFI; IFI; RMSEA (1.000; 1.000; 0.094). CFI = Comparative fit index; IFI = incremental fit index; RMSEA = root mean square error of approximation.

Figure 2 illustrates the path analysis examining the direct and indirect effects of the Child Adolescent Bullying Scale (CABS) on the Strengths and Difficulties Questionnaire (SDQ), with social communication challenges acting as a mediating variable among 150 Saudi children with Autism Spectrum Disorder (ASD). The model fit parameters indicate a reasonable fit, with Comparative Fit Index (CFI) and Incremental Fit Index (IFI) both at 1.000 and a Root Mean Square Error of Approximation (RMSEA) of 0.094, suggesting a well-fitting model. The significant chi-square value (χ^2^ = 11.812, *p* < 0.001) supports the overall model’s significance. The analysis reveals that bullying has a direct effect on the SDQ scores. At the same time, social communication challenges mediate this relationship, indicating that the negative impact of bullying on emotional and behavioral difficulties is partially transmitted through social communication issues. The terms “e1” and “e2” denote the error terms associated with the latent variables “Social Communication” and “Strengths and Difficulties,” respectively. These error terms account for the variance in each construct that is not captured by the model. The “1” indicates a fixed value for the error terms, which is commonly set in structural equation modeling to ensure proper identification of the model.

Table 5 presents the direct and indirect effects of variables related to bullying and social communication challenges on the Strengths and Difficulties Questionnaire (SDQ) scores among 150 Saudi children with Autism Spectrum Disorder (ASD). The direct effect of bullying on social communication challenges is reported as 0.102, with a critical ratio (C.R.) of 3.930, indicating a statistically significant relationship (*p* < 0.001). This suggests that experiences of bullying significantly contribute to social communication difficulties. Additionally, the table shows a direct effect of social communication challenges on SDQ scores of 0.261, with an indirect effect of 0.027, yielding a C.R. of 3.090 (*p* = 0.002). This indicates that social communication challenges not only directly impact emotional and behavioral difficulties but also mediate some of the effects of bullying. Finally, the direct effect of bullying on the SDQ scores is 0.069, with a C.R. of 2.446 (*p* = 0.014), reinforcing that bullying has a measurable impact on the overall difficulties experienced by these children.

## 4. Discussion

Bullying is a pervasive issue that significantly impacts the well-being of children, particularly those with Autism Spectrum Disorder (ASD). In Saudi Arabia, children with ASD often experience heightened vulnerability to bullying due to their social communication challenges, which can exacerbate their emotional and behavioral difficulties [45]. This study aims to explore the intricate relationships between bullying, social communication challenges, and the emergence of mental health problems among Saudi children with ASD. By examining how these factors interact, the study seeks to uncover the underlying mechanisms that contribute to the mental health challenges faced by this population.

The current study findings indicate a concerning level of bullying experienced by these children, which is consistent with existing literature that highlights the vulnerability of children with ASD to bullying due to their social communication deficits [46]. This prevalence not only affects their immediate social interactions but can also have long-term implications for their emotional well-being and development, emphasizing the need for culturally relevant interventions. The significant difficulties reflected in the social communication challenges underscore the core characteristics of ASD, where impaired social skills can lead to difficulties in forming friendships and engaging in typical peer interactions [47].

Such challenges can exacerbate feelings of loneliness and social isolation, further complicating the emotional landscape for these children [48]. In Saudi Arabia, cultural norms that emphasize conformity and peer acceptance may further marginalize children with ASD, making them targets for bullying [27]. The schooling system, which often lacks tailored support for neurodiverse students, can exacerbate these challenges, as teachers and peers may not fully understand or accommodate their unique needs [49]. Additionally, family expectations regarding social behavior can place further pressure on these children, complicating their social interactions even more. Research has indicated that social communication deficits are closely linked to increased emotional problems, which can manifest in anxiety and depression [50].

The results from the Strengths and Difficulties Questionnaire reveal various emotional and behavioral difficulties among the participants. The elevated scores in emotional problems denote a high prevalence of mental health concerns, which aligns with findings from studies indicating that children with ASD are at a greater risk for emotional disorders compared to their neurotypical peers [50,51]. Furthermore, the relatively low scores in pro-social behaviors indicate that these children may struggle to engage positively with their peers, which could further perpetuate their social difficulties and contribute to a cycle of isolation and emotional distress [11].

Moreover, the significant positive correlation between bullying and social challenges indicates that children who experience higher levels of bullying also face greater difficulties in social communication. This finding emphasizes the need to consider the sociocultural factors that may influence the experiences of children with ASD in Saudi Arabia. The interplay between bullying and social communication challenges suggests that interventions should focus on enhancing social skills while simultaneously addressing bullying prevention. This finding aligns with existing literature that found that children with ASD are particularly vulnerable to bullying due to their social skills deficits, which can exacerbate their challenges in peer interactions [52]. The negative impact of bullying on social skills can lead to a cycle of isolation and increased vulnerability to emotional distress.

In addition, the correlations between bullying and both conduct and emotional problems further underscore the detrimental effects of bullying on mental health. Children who are bullied are more likely to exhibit behavioral issues and emotional difficulties, which can manifest as anxiety, depression, or aggressive behavior [53]. This is particularly concerning given that emotional problems are not only prevalent but also strongly correlated with overall difficulties as measured by the Strengths and Difficulties Questionnaire. This reinforces the need for a holistic approach that encompasses both emotional and behavioral challenges, as neglecting one may exacerbate the other. Such emotional challenges can hinder academic performance and social integration, making it essential to address these issues comprehensively. Furthermore, the strong positive correlations among social challenges, emotional problems, and peer problems highlight the interconnected nature of these variables. Difficulties in social communication can lead to increased emotional distress and challenges in developing peer relationships, creating a feedback loop that reinforces the impact of bullying and social isolation [50].

The regression analysis of the current study identifies both the Child Adolescent Bullying Scale (CABS) and social communication challenges as significant predictors of SDQ scores. The findings indicate that higher levels of bullying are associated with increased emotional and behavioral difficulties, highlighting the detrimental impact of bullying on children with ASD. This aligns with the theoretical framework of the ecological systems theory, which posits that individual behavior is influenced by various interacting systems, including family, school, and community [54]. Within this framework, the direct effects of bullying on emotional and behavioral outcomes can be understood as a result of negative interactions within these systems, where environmental stressors contribute to the emotional distress experienced by children with ASD [55]. This aligns with existing literature that emphasizes the vulnerability of children with ASD to bullying, which can exacerbate their emotional and behavioral challenges [51]. The analysis also reveals that social communication challenges significantly contribute to the variability in SDQ scores.

Importantly, the mediation analysis indicates that the mediation of social communication challenges is partial, meaning that while bullying has a direct effect on emotional and behavioral difficulties, social communication challenges also play a significant role in exacerbating these effects. This highlights the importance of addressing both bullying and social communication skills in interventions. Children with ASD often struggle with social interactions, which can lead to increased emotional distress and behavioral issues [50]. This finding is consistent with research indicating that social communication deficits are closely linked to higher rates of emotional problems in children with ASD [56]. Thus, addressing these communication challenges is vital for improving mental health outcomes, particularly in a culturally sensitive manner that resonates with Saudi Arabian societal norms.

The model explains a modest portion of the variance in SDQ scores, indicating that while bullying and social communication challenges are significant factors, other variables may also play a role in influencing the mental health outcomes of children with ASD. This indicates that a broader range of factors, including family dynamics, cultural context, and community support, should be considered to fully understand and address the complexities of mental health in this population. The practical implication of these findings is that interventions should not only focus on reducing bullying behaviors but also enhance social communication skills, thereby providing children with the tools to navigate social interactions more effectively. The statistical significance of the model reinforces the importance of these findings in guiding future research and intervention strategies aimed at improving the well-being of children with ASD.

Additionally, the model exhibits reasonable fit parameters, confirming the robustness of the identified relationships. The direct effect of bullying on SDQ scores highlights the significant emotional and behavioral challenges faced by these children, a finding consistent with existing literature that underscores the vulnerability of children with ASD to bullying and its adverse impacts on their mental health [52]. Notably, the mediation of social communication challenges indicates that difficulties in social interactions exacerbate the negative effects of bullying, suggesting that enhancing social communication skills could serve as a protective factor. This aligns with previous research advocating for culturally sensitive interventions that improve social skills among children with ASD to mitigate the emotional toll of bullying [6,57].

## 5. Limitations of the Study

Despite its important findings, the study has several limitations. Firstly, the cross-sectional design limits the ability to establish causal relationships between bullying, social communication challenges, and mental health outcomes. Secondly, the reliance on self-reported data may introduce biases, as participants might not accurately reflect their experiences with bullying or mental health issues. Additionally, while the sample size of 150 participants is reasonable, a larger and more diverse sample could improve the generalizability of the findings. Furthermore, the sample was recruited from a specific source, a single nonprofit institution, which may not represent the broader population of children with ASD in Saudi Arabia. The cultural context of Saudi Arabia may also impact the applicability of the results to other regions, where social dynamics and bullying behaviors may differ. Lastly, the study’s focus on specific validated instruments limits the exploration of other potential contributing factors, such as family dynamics or socioeconomic status, which could further elucidate the mental health challenges faced by these children. Additionally, the lack of longitudinal data hinders our understanding of how these relationships may develop over time. Furthermore, important variables such as support systems and access to mental health resources were not assessed, which could provide a more comprehensive view of the challenges faced by these children.

## 6. Conclusions

This study highlights the complex relationship between bullying, social communication challenges, and the mental health issues faced by Saudi children with Autism Spectrum Disorder (ASD). While existing literature has established connections among these factors, our research brings attention to the unique sociocultural context of Saudi Arabia, which may shape the experiences of children with ASD differently than in other regions. The findings indicate that these children are particularly vulnerable to bullying, which significantly worsens their social challenges and contributes to various emotional and behavioral problems. These results underline the urgent need for culturally informed early detection and intervention strategies that address both bullying and social skills deficits. By fostering an encouraging environment and enhancing social communication skills, we can improve the overall mental health and well-being of children with ASD, ultimately aiding their integration and quality of life.

## 7. Recommendations

Considering the study’s findings, several recommendations can be made. First, schools and communities should develop comprehensive anti-bullying initiatives tailored to the specific needs of children with ASD, considering the unique sociocultural dynamics of Saudi Arabia while promoting awareness and empathy among peers. Additionally, enhancing social skills training is essential, as interventions designed to improve communication abilities can help children with ASD build better relationships and resilience against bullying. Regular mental health monitoring should become standard practice for children with ASD, ensuring that any emerging emotional or behavioral issues are addressed promptly. Engaging parents through training programs can empower them to recognize signs of bullying and effectively support their children. Finally, further research should explore longitudinal designs to assess the long-term impacts of bullying on children with ASD and examine other sociocultural factors that may affect mental health outcomes.

## 8. Implications for Practice

The findings from this study hold significant implications for clinical practice, particularly for mental health professionals, educators, and caregivers working with children with ASD. Understanding the dynamics between bullying, social communication difficulties, and mental health in the context of Saudi Arabia can inform the development of intervention strategies specifically tailored to meet the needs of these children. Clinicians can leverage these insights to design therapeutic approaches that focus on enhancing social skills and providing emotional support, thereby addressing the unique challenges faced by children with ASD in this cultural environment. Furthermore, this study emphasizes the importance of a multidisciplinary approach, fostering collaboration among mental health professionals, educators, and families. By working together, we can create a supportive environment that promotes the mental health and well-being of children with ASD, ultimately leading to improved social integration and quality of life.

## Figures and Tables

**Table 1:** Sociodemographic characteristics (n = 150).

Sociodemographic Characteristics	No.	%
**Age (years)**		
<5	6	4.0
5–<10	77	51.3
10–<15	56	37.3
≥15	11	7.3
Mean ± SD	9.20 ± 3.33	
**Gender**		
Male	110	73.3
Female	40	26.7
**Age of the child at the onset of the disease**		
Since birth	11	7.3
<2	4	2.7
2–<4	113	75.3
4–<6	20	13.3
≥6	2	1.3
Mean ± SD	2.56 ± 1.18	
**Duration of disease**		
<5	13	8.7
5–<10	115	76.7
≥10	22	14.7
Mean ± SD	7.71 ± 2.44	
**Presence of comorbid disease**		
Yes	20	13.3
No	130	86.7
**If yes (n = 20)**		
Double disability	2	10.0
Visual disability	2	10.0
ADHD	2	10.0
Other	14	70.0
**Did the child receive any treatment?**		
Yes	150	100.0
No	0	0.0
**If yes (n = 150)**		
Pharmacological treatment	10	6.7
Psychiatric or behavior treatment	103	68.7
Speech treatment	43	28.7
Pharmacological and speech	5	3.3
Pharmacological, Psychiatric, or behavioral treatment	25	16.7
**Family history of autism**		
Yes	42	28.0
No	108	72.0
**Frequency of bullying exposure**		
Not bullied at all	41	27.3
Not much	84	56.0
Quite a lot	19	12.7
A lot	6	4.0
**Presence of a perceived power differential between the youth and the perpetrator**		
More power	16	10.7
Moderate power	122	81.3
Less power	12	8.0

**Table 2:** Descriptive analysis of the study variables (n = 150).

Variables	Total Score		Mean Percent Score	
	Mean	±SD	Mean	±SD
**Child Adolescent Bullying Scale (CABS)**	**46.37**	**18.65**	**32.96**	**23.31**
**Social Communication Challenges**	**17.45**	**6.23**	**44.73**	**15.98**
**Strengths and Difficulties Questionnaire (SDQ)**	**21.35**	**6.57**	**42.71**	**13.13**
Pro-social	2.27	2.14	22.73	21.42
Conduct problems	4.05	2.26	40.47	22.59
Emotional problems	10.14	4.50	50.70	22.52
Peer problems	4.89	2.48	48.93	24.83

**Table 3:** Correlation between the studied variables (n = 150).

		Bullying	Social Communication Challenges	Pro-Social	Conduct Problems	Emotional Problems	Peer problems	Overall SDQ
**Social Communication Challenges**	**r**	0.306 *						
	***p***	<0.001 *						
Pro-social	**r**	−0.157	−0.305 *					
	***p***	0.055	<0.001 *					
Conduct problems	**r**	0.188 *	0.177 *	−0.395 *				
	***p***	0.021 *	0.030 *	<0.001 *				
Emotional problems	**r**	0.247 *	0.336 *	−0.604 *	0.532 *			
	***p***	0.002 *	<0.001 *	<0.001 *	<0.001 *			
Peer problems	**r**	0.236 *	0.308 *	−0.499 *	0.341 *	0.585 *		
	***p***	0.004 *	<0.001 *	<0.001 *	<0.001 *	<0.001 *		
**Overall SDQ**	**r**	0.272 *	0.308 *	−0.413 *	0.709 *	0.894 *	0.734 *	
	***p***	0.001 *	<0.001 *	<0.001 *	<0.001 *	<0.001 *	<0.001 *	

r: Pearson correlation coefficient *: Statistically significant at *p* ≤ 0.05.

**Table 4:** Multivariate linear regression analysis for factors affecting the Strengths and Difficulties Questionnaire (SDQ) (n = 150).

Variable	B	Beta	t	*p*	95% CI	
					LL	UL
**Child–Adolescent Bullying Scale (CABS)**	0.069	0.196	2.430 *	0.016 *	0.013	0.125
**Social Communication Challenges**	0.261	0.248	3.069 *	0.003 *	0.093	0.429
R^2^ = 0.130, Adjusted R^2^ = 0.118, F = 10.977 *, *p* < 0.001 *						

F, *p*: f- and *p*-values for the model. R^2^: Coefficient of determination. B: Unstandardized coefficients. Beta: Standardized coefficients. t: *t*-test of significance. LL: Lower limit. UL: Upper limit. *: Statistically significant at *p* ≤ 0.05.

**Table 5:** Direct and indirect effect.

Variable 1	Variable 2	Direct Effect	Indirect Effect	C.R	*p*-Value
**Social Communication Challenges**	Bullying	0.102		3.930 *	<0.001 *
**Strengths and Difficulties**	Social Challenges	0.261	0.027	3.090 *	0.002 *
**Strengths and Difficulties**	Bullying	0.069		2.446 *	0.014 *

*: Statistically significant at *p* ≤ 0.05.

## Data Availability

The datasets used or analyzed in this study are available from the corresponding author upon request.
